# Epigenetic modification of Castor zinc finger 1 (CASZ1) is associated with tumor microenvironments and prognosis of clear cell renal cell carcinoma

**DOI:** 10.1097/JS9.0000000000002070

**Published:** 2024-09-04

**Authors:** Fei Li, Jiayu Liang, Xin Wei

**Affiliations:** Department of Urology, Institute of Urology, West China Hospital, Sichuan University, Chengdu City, Sichuan Province, People’s Republic of China

**Keywords:** CASZ1, clear cell renal cell carcinoma, methylation, tumor microenvironment

## Abstract

**Background:**

Clear cell renal cell carcinoma (ccRCC) represents the predominant and remarkably diverse form of renal cell carcinoma. The involvement of the Castor zinc finger 1 (CASZ1) gene in adverse prognostic outcomes has been observed across different cancer types. Nevertheless, the specific altered activities and associated multi-omics characteristics of CASZ1 in ccRCC remain unelucidated.

**Method:**

In order to explore the expression of CASZ1, evaluate its prognostic significance, and aid in the therapeutic decision-making process for patients with ccRCC, The Cancer Genome Atlas (TCGA), Gene expression omnibus (GEO), and The Human Protein Atlas (HPA) databases were utilized to gather data on clinicopathological data, prognostic information, genomic, methylomic and immunomic data. Additionally, the Genomics of Drug Sensitivity in Cancer (GDSC) database provided information on drug sensitivity.

**Results:**

CASZ1 expression was found to be significantly reduced in ccRCC and was associated with unfavorable pathological characteristics and a bleak prognosis. Diminished CASZ1 mRNA levels were notably correlated with heightened cytosine-phosphate-guanine (CpG) methylation, indicating a poorer prognosis for patients with increased methylation. Examination of RNA-seq data from TCGA indicated that the CASZ1-high expression subgroup displayed heightened immune cell infiltration and increased expression of immune checkpoint markers, potentially suggesting a more favorable response to immunotherapy. Furthermore, data from the GDSC database indicated that the CASZ1-low expression subgroup might exhibit greater sensitivity to anti-angiogenetic treatments, such as Sunitinib and Axitinib.

**Conclusions:**

These results indicate that CASZ1 may function as a biomarker for distinguishing various tumor microenvironment phenotypes, predicting prognosis, and assisting in treatment decisions for individuals with ccRCC.

## Background

Highlights

- CASZ1 expression was found to be significantly reduced in ccRCC and was associated with unfavorable pathological characteristics and a bleak prognosis.
- Diminished CASZ1 mRNA levels were notably correlated with heightened CpG methylation, indicating a poorer prognosis for patients with increased methylation.
- CASZ1-high subgroups may be more suitable for PD-1, PD-L1 pathway blockade therapy.
- Patients with ccRCC alongside CASZ1-low expression may benefit from sunitinib and axitinib.

Renal cell carcinoma (RCC) is the most common type of cancerous kidney tumor, constituting up to 85% of cases, and its global incidence has been increasing over the past few decades, with the highest annual mortality rate among urological carcinomas^1–4^. Based on its varied morphologies and distinct driver gene modifications, RCC has been divided into a minimum of 12 subgroups, and these comprise papillary renal cell carcinoma (pRCC), chromophobe renal cell carcinoma (chRCC), and clear cell renal cell carcinoma (ccRCC), which collectively account for about 90% of cases of RCC^5^. Though health tests and imaging technology have advanced, a growing number of RCC cases are being diagnosed early and are amenable to curative radical surgery. However, ~30–40% of RCC cases progress to metastatic disease, necessitating systemic therapy^6^. Research indicates that around 20–30% of patients exhibit metastatic symptoms at the time of diagnosis, with a mere 12% 5-year overall survival rate^7^.

Advancements in molecular understanding have led to the application of new immunotherapies and targeted treatments, significantly improving the prognosis of metastatic renal cell carcinoma. As a result, the outlook for metastatic renal cell carcinoma has notably enhanced, leading to a median overall survival that has risen from under 1 year to more than 4 years^8,9^. Nevertheless, the ongoing dilemma between tumor diversity and personalized treatment approaches somewhat hampers the effectiveness of these novel medications. Consequently, there is a pressing need to identify new biomarkers that can predict the prognosis of renal cell carcinoma and optimize treatment decisions. Molecular subtypes of RCC that have been documented include PD-L1 and PBRM1^10,11^. However, the use of these genes as accurate indicators to categorize ccRCC patient treatment approaches is a topic of debate^12,13^. Thus, the search for novel and potent biomarkers is imperative.

Castor zinc finger 1 (CASZ1) is a transcription factor initially known for its role in neural fate determination and critical functions in neural and cardiac developmental processes^14,15^. Numerous studies have indicated that CASZ1 facilitates vascular assembly and morphogenesis by controlling endothelial cell contractility and adhesion^16,17^. Recent studies have highlighted the increasing recognition of CASZ1’s importance in tumorigenesis. It has been shown to regulate T helper cell plasticity and play a crucial role in autoimmune inflammation^18^. Moreover, CASZ1’s involvement in tumor progression has been reported, with implications for neuroblastoma and hepatocellular carcinoma^19–22^. Conversely, CASZ1’s high expression has been linked to cell migration and invasion in epithelial ovarian cancer, indicating diverse expression patterns and functions in various human cancers^23^. Some research has suggested that CASZ1 expression is down-regulated and associated with poor prognosis in ccRCC^24^. Therefore, this study aims to investigate CASZ1 expression, analyze its prognostic value, and contribute to therapeutic decision-making for ccRCC patients.

## Methods

### RNA and protein expression analysis

Patients who fulfilled the following selection criteria were considered: (a) diagnosed with histologically confirmed papillary renal cell carcinoma (pRCC), chromophobe renal cell carcinoma (chRCC), and clear cell renal cell carcinoma (ccRCC); (b) available overall survival (OS) data and RNA-sequencing data. (c) can be retrieved and acquired from public databases or our institution. This work has been reported in line with the REMARK criteria^25^.

Using the TCGA (The Cancer Genome Atlas) database, we initially evaluated the variations in CASZ1 gene expression between adjacent normal tissues and malignant tissues across a range of malignancies. TIMER (Tumor Immune Estimation Resource) was then used to show the data^26,27^. To search for patterns in RNA expression In this cohort, this analysis included 532 tumor tissue samples and 72 adjacent normal tissue samples for kidney renal clear cell carcinoma (ccRCC), 290 tumor tissue samples and 32 adjacent normal tissue samples for kidney renal papillary cell carcinoma (pRCC), and 65 tumor tissue samples and 25 adjacent normal tissue samples for kidney chromophobe (chRCC). To verify the results of the TCGA database, we then acquired and employed three microarray datasets—GSE53757, GSE40435, and GSE66272—from the GEO (Gene Expression Omnibus) database in order to confirm the expression levels of CASZ1 in ccRCC. Each dataset included a minimum of twenty human ccRCC and surrounding normal tissues^28^. The significance level was determined by using the Wilcox rank sum test (*P*<0.05 indicated statistical significance).

Furthermore, making use of three normal tissue samples and twelve tumor tissue samples from HPA(The Human Protein Atlas), immunohistochemistry was employed to confirm CASZ1 expression at the protein level^29^. For staining, a 1:90 dilution of Atlas Antibodies’ HPA028222 antibody was used. The immunohistochemical scoring and its annotations were provided by the HPA.

Finally, 20 formalin-fixed, paraffin-embedded (FFPE) tissues from our own institution were used for immunohistochemistry to again support the results of the above analysis. Under the ethical guidelines as required by the Declaration of Helsinki, informed consent was provided by each patient, and the research protocol was approved by the West China Hospital of Sichuan University Biomedical Research Ethics Committee. Briefly, sections were deparaffinized and rehydrated through different concentrations of ethanol. Then, tissue antigen retrieval was performed with a citrate buffer solution. After being placed in 3% H_2_O_2_ and blocking buffer at room temperature, slides were incubated with anti-CASZ1 antibody (SC-398303,1:50) overnight at 4°C. The stained tissue slides were scanned by a digital trinocular camera microscope system(MOTICCHINAGROUPCO.,LTD.;BA400Digital), and the positive area percentage for each image were calculated by a data image analysis system (Indica labs, U.S.A; Halo 101-WL-HALO-1). We then used the Wilcoxon rank sum test for statistical analysis and the ggplot2 package to visualize the data.

### Clinicopathological and prognostic analysis

We initiated an assessment of the association between CASZ1 mRNA expression and various clinical features of ccRCC, pRCC, and chRCC. This encompassed the analysis of pT stage, pN stage, metastatic status, and tumor grading. RNA-sequencing expression profiles and corresponding clinical information for ccRCC, pRCC, and chRCC were downloaded from the TCGA dataset. Statistical analysis and ggplot2 (v3.3.2) were completed using R program v4.0.3, *P* value less than 0.05 was considered statistically significant. Subsequently, we conducted an overall survival (OS) analysis to evaluate the potential association between the expression status of the CASZ1 gene and the prognosis of ccRCC, pRCC, and chRCC, respectively. OS is defined as the time from randomization to death. Any patients lost to follow-up or still alive at the time of evaluation are censored. We performed optimal grouping cut-off selection using the surv_cutpoint function in the survminer package. Then, we used the survival package to perform survival regression and proportional hazards assumption testing. The ggplot2 and survminer software were used to visualize the results.

### Epigenetic modification analysis

Initially, we analyzed the CpG-aggregated methylation value of CASZ1 among a series of malignant tumors through SMART^30^. Subsequently, after controlling for tumor purity, we investigated the relationship between the CASZ1 gene and numerous genes, including DNMT1, TRDMT1, DNMT3A, DNMT3B, TET1, TET2, TET3, MBD1, MBD2, MBD3, MBD4 and MECP2 implicated in DNA methylation by TIMER^31^. The purity-adjusted partial Spearman’s rho value was displayed as a heatmap, with *P* less than 0.05 being deemed statistically significant. The correlation was verified, using the Spearman rank test. Next, we looked at the MethSurv-based methylation status of a number of probes in the TCGA ccRCC cohort’s CASZ1 DNA^32^. Furthermore, using SMART and the Pearson method to compute the correlation coefficient, we examined the particular associations between the degree of methylation and CASZ1 expression in ccRCC^30^. To find the probes where CASZ1 is hyper-methylated, we set the adjusted *P* value for the cut-off at 0.01 and the cut-off value of the beta value at 0.25. Next, we turned our attention to these probes and looked into how the degree of methylation in ccRCC in both the tumor and normal tissues differed. Finally, we examined the survival difference of these probes between high-methylation and low-methylation subgroups. All the above analyses were conducted and presented by SMART.

### Functional and pathway enrichment analysis

To learn more about the biological processes and signaling pathways connected to the expression of the CASZ1 gene, we initially utilized the “Limma” package to assess mRNA differential expression between the CASZ1-low and CASZ1-high subgroups. A volcano plot was generated to visualize significantly over-expressed and under-expressed mRNAs, with adjusted *P* less than 0.05 and fold change=1.3 set as the cut-off values. Given the abundance of distinct genes, a heatmap was also created to display the top 50 genes with up- and down-regulation, representing expression trends in ccRCC samples. Subsequently, using the TCGA’s ccRCC transcriptome data, we performed Kyoto Encyclopedia of Gene and Genomes (KEGG) pathway enrichment analysis and Gene Ontology (GO) enrichment analysis. In order to enrich the KEGG pathway and examine the GO function of differential genes between the CASZ1-low and CASZ1-high subgroups, we used the “ClusterProfiler” package. A pathway was deemed to be enriched if *P* less than 0.05 or FDR less than 0.05.

### Immune infiltration and immune checkpoint analysis

We compared immune cell score distributions between CASZ1-low and CASZ1-high subgroups using the xCELL algorithm, utilizing the “immunedeconv” package, in order to investigate potential associations between CASZ1 expression and the immunological conditions of ccRCC in the tumor microenvironment (TME). A heatmap was generated, then, to assess the significance of the two groups, the Wilcox test was employed. In addition, the proportions of various immune cells in microenvironment of ccRCC were calculated and exhibited. Furthermore, the immune checkpoint genes, SIGLEC15, TIGIT, CD274, HAVCR2, PDCD1, CTLA4, LAG3, and PDCD1LG2, that are expressed differently in the CASZ1-low and CASZ1-high subgroups were extracted using the “ggplot2” program.

### Analysis of potential therapeutic strategies

Firstly, we gathered clinical data of the TCGA’s ccRCC cohort that matched RNA-sequencing results. The GDSC (Genomics of Drug Sensitivity in Cancer) was then utilized to forecast the ccRCC samples’ targeted therapeutic response. Using IC50, We evaluated the effect of TKI medicines on the treatment response between the CASZ1-low and CASZ1-high subgroups, specifically Sunitinib, Pazopanib, and Axetinib. With all parameters set to default, we used ridge regression to estimate the IC50 of each sample while controlling for the tissue type and batch effect of battle. Additionally, the mean value served as our summary of the recurrent gene expression.SIGLEC15, TIGIT, CD274, HAVCR2, PDCD1, CTLA4, LAG3 and PDCD1LG2 were selected to be immune-checkpoint-relevant transcripts and the expression values of these eight genes were extracted. Then, the differential expression of the eight immunological checkpoints between the CASZ1-low and CASZ1-high subgroups were compared. All the above analysis methods were implemented by the ggplot2 R package and pheatmap R package. Finally, using the TIGER tool^33^, we analyzed data from a pd-1 treatment dataset, RCC-GSE67501_anti-PD-1^34^, to further validate and support the conclusions.

## Results

### RCC showed a markedly reduced expression of CASZ1

**Figure 1:**
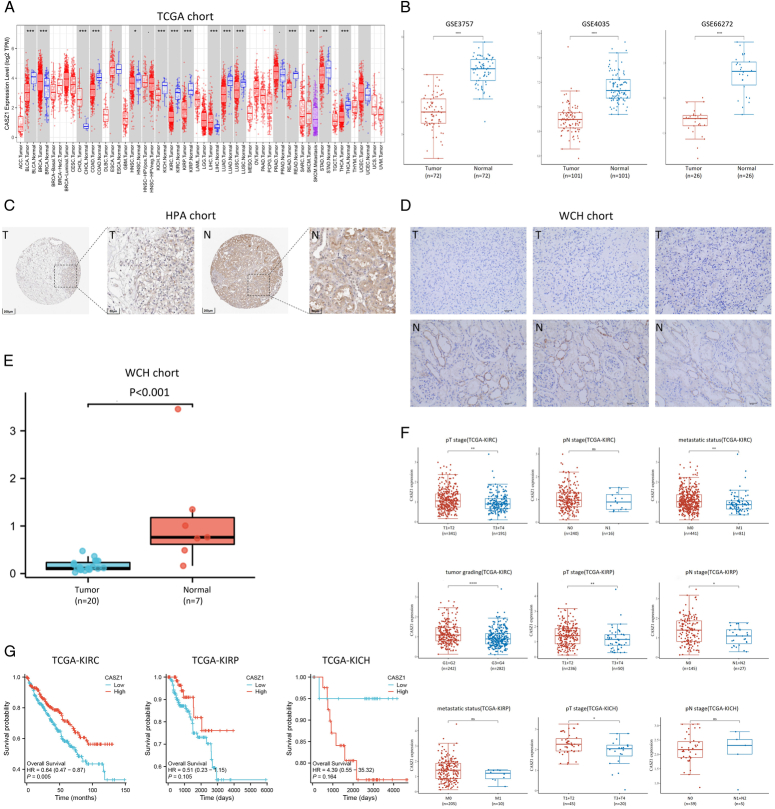
Castor zinc finger 1 (CASZ1) mRNA and protein expression was lower in renal cell carcinoma (RCC) tissues than that in normal kidney tissues. Low CASZ1 expression was associated with several clinicopathological characteristics and poor prognosis in clear cell renal cell carcinoma (ccRCC). (A) CASZ1 mRNA expression in tumor and normal tissues from pan-cancer data of The Cancer Genome Atlas (TCGA). *P<0.05, **P<0.01, ***P<0.001. (B) CASZ1 mRNA expression in tumor and normal tissues from ccRCC obtained from Gene Expression Omnibus (GEO) database, including GSE53757, GSE40435 and GSE66272. ****P<0.0001. (C) Representative immunohistochemical staining of CASZ1 in normal kidney tissue and ccRCC tissue from HPA (The Human Protein Atlas). (D) Representative immunohistochemical staining of CASZ1 in adjacent normal tissues and ccRCC tissue from our institution. (E) The percentage of positive area of tumor tissue was significantly lower than that of adjacent normal tissue. (F) CASZ1 mRNA expression was associated with pT stage, metastatic status and tumor grading in ccRCC, but not with pN stage. CASZ1 mRNA expression was associated with pT stage and pN stage in papillary renal cell carcinoma (pRCC), but not with metastatic status. CASZ1 mRNA expression was associated with pT stage in chromophobe renal cell carcinoma (chRCC), but not with pN stage.*P<0.05, **P<0.01,***P<0.001, ****P<0.0001. (G) Kaplan–Meier analysis of the association between CASZ1 expression and OS in ccRCC, pRCC, chRCC.

Initially, we assessed the differential expression of CASZ1 in tumor and normal tissues at pan-cancer RNA level, using data from the TCGA database. Compared to corresponding normal tissues, CASZ1 expression was notably reduced in all three major subtypes of RCC, with a *P* value less than 0.001 (Fig. 1A). Three microarray datasets from the GEO database were used to verify the CASZ1 expression pattern in ccRCC tumor specimens: GSE53757 (*n*=72), GSE40435 (*n*=101), and GSE66272 (*n*=26). All of these datasets showed a *P* value less than 0.001 (Fig. 1B).

Then, we examined the protein-level expression pattern of CASZ1 in RCC and adjacent normal tissues using paraffin-embedded samples from the HPA database. Through the use of immunohistochemical staining, it was possible to determine that, although CASZ1 protein was undetectable in all 12 of the ccRCC tumor tissues, it was visible in the membrane, cytoplasm, and nucleus of the renal tubules in each of the three normal tissues with moderate intensity and 75–25% quantity (Fig. 1C). Our institution’s experimental results is shown in Tables 1 and 2. It indicated that the majority of the tumor tissue regions show no positive expression (Fig. 1D), with the positive area percentage significantly lower than that in the adjacent normal tissues (Fig. 1E).

**Table 1:** CASZ1 (tumor).

Sample number	Positive area percentage	Average value	Sample number	Positive area percentage	Average value
ccRCC-1	0.730973	0.4750	ccRCC-12	0.083501	0.1038
	0.491439			0.08206	
	0.20262			0.145938	
ccRCC-2	0.290638	0.1161	ccRCC-13	0.101133	0.0814
	0.031366			0.030856	
	0.026367			0.112315	
ccRCC-3	0.078751	0.1005	ccRCC-14	0.120242	0.1045
	0.124064			0.049071	
	0.098587			0.144313	
ccRCC-4	0.25802	0.2696	ccRCC-15	0.025476	0.0238
	0.108295			0.017101	
	0.442411			0.028789	
ccRCC-5	0.268943	0.2681	ccRCC-16	0.084309	0.1894
	0.385349			0.366561	
	0.150118			0.117233	
ccRCC-6	0.074574	0.1055	ccRCC-17	0.384222	0.2689
	0.08133			0.220263	
	0.160544			0.202237	
ccRCC-7	0.103178	0.1325			
	0.118752				
	0.17568				
ccRCC-8	0.078813	0.0788	ccRCC-18	0.075755	0.0758
ccRCC-9	0.087315	0.0873	ccRCC-19	0.060185	0.0602
ccRCC-10	0.364906	0.3649	ccRCC-20	0.13991	0.1399
ccRCC-11	0.221253	0.2213			

CASZ1, Castor zinc finger 1; ccRCC, clear cell renal cell carcinoma.

**Table 2:** CASZ1 (adjacent normal tissues).

Sample number	Positive area percentage	Average value	Sample number	Positive area percentage	Average value
ccRCC-8	0.149412	0.1624	ccRCC-18	0.566586	0.4890
	0.175365			0.4114	
ccRCC-9	1.267286	1.3484	ccRCC-19	0.634853	0.7626
	1.429506			0.890379	
ccRCC-10	3.48487	3.4564	ccRCC-20	0.473486	0.7408
	3.428021			1.008112	
ccRCC-11	0.956478	1.0070			
	1.057551				

CASZ1, Castor zinc finger 1; ccRCC, clear cell renal cell carcinoma.

Downregulation of CASZ1 was observed in renal cancer tissues at the protein level, which was in line with the mRNA-level findings.

### A poor prognosis and unfavorable clinicopathological characteristics were linked to low CASZ1 expression

We examined CASZ1 dysregulation’s possible functional involvement in RCC by examining clinicopathological characteristics such as tumor grading (ISUP grading), metastatic status, and pT and pN stages. When based on RCC histological types stratification, the mRNA expression level of CASZ1 showed an inverse correlation with clinicopathological characteristics (Fig. 1F). The expression of CASZ1 in ccRCC exhibited an inverse relationship with the tumor grade (*P*<0.0001), metastatic status (*P*<0.01), and pT stage (*P*<0.01). The pT (*P*<0.01) and pN (*P*<0.05)stages of pRCC were inversely connected with CASZ1 expression. In chRCC, the expression of CASZ1 is negatively correlated with pT staging (*P*<0.05). Lastly, an analysis of the relationship between CASZ1 mRNA expression and overall survival (OS) from the TCGA database revealed that the CASZ1-low expression subgroup in ccRCC had a poor OS, compared to the CASZ1-high expression subgroup (*P*=0.05), but CASZ1 expression had no effect on OS in pRCC and chRCC (Fig. 1G).

### CASZ1 downregulation in ccRCC was closely associated with hypermethylation

**Figure 2:**
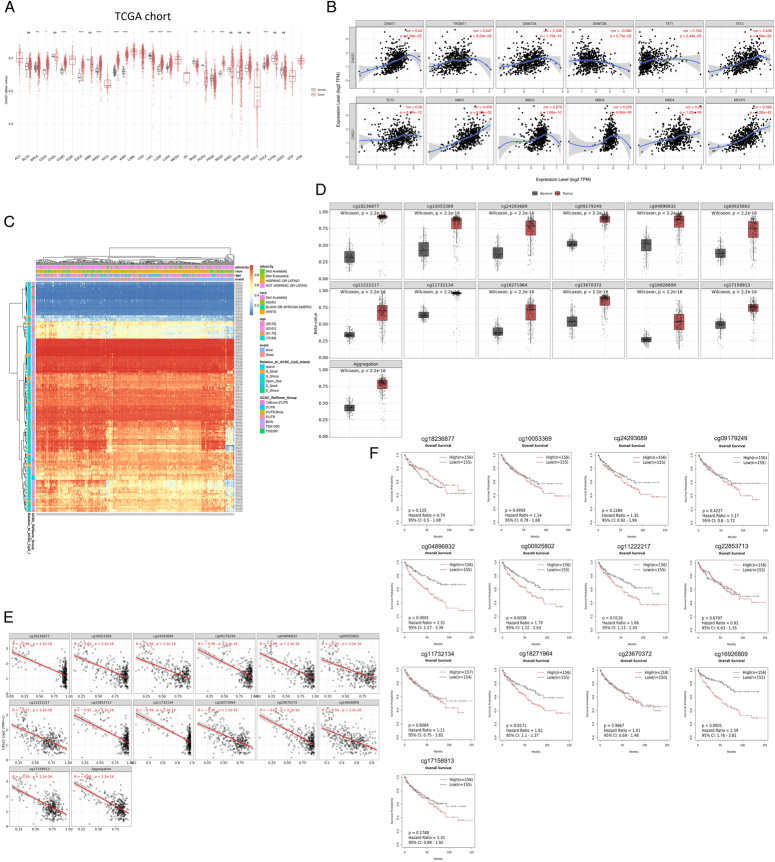
Castor zinc finger 1 (CASZ1) expression was associated with DNA methylation modification in clear cell renal cell carcinoma (ccRCC). (A) CpG-aggregated methylation value of CASZ1 in tumor and normal tissues from pan-cancer data of The Cancer Genome Atlas (TCGA). *P<0.05, **P<0.01, ***P<0.001,****P<0.0001. (B) The correlation of CASZ1 expression and the expression of DNA methylation-related genes. (C) The association of methylation level with gene subregions. (D) The DNA methylation level of different probes between normal and tumor tissues. (E) The association between CASZ1 expression and these probes’ methylation levels in ccRCC. (F) The relationship between the prognosis and the methylation levels of these probes in ccRCC.

We examined the TCGA database to seek out the potential function of regulation of DNA methylation in CASZ1 downregulation. The results showed that KIRC and KIRP tumor tissues had notably higher levels of CpG-aggregated methylation of CASZ1 than did normal tissues, respectively, *P* less than 0.0001. (Fig. 2A). Furthermore, we discovered a favorable correlation between the expression of several DNA methylation-related enzymes and the expression of the CASZ1 gene in ccRCC, including TET2 (*r*=0.439, *P*<0.001), TET3 (*r*=0.29, *P*<0.001) and TET1 (*r*=0.182, *P*<0.001) (Fig. 2B). The TET family enzymes have the ability to oxidize the 5-methylcytosine group, converting it to 5-hydroxymethylcytosine, thereby promoting the process of DNA demethylation, making DNA molecules more prone to unwinding, facilitating the binding of transcription factors, and regulating gene expression^35^. Thus when the expression of these enzymes is up-regulated, the level of gene DNA methylation modification is down-regulated, leading to enhanced gene transcriptional activity, upregulating gene expression. Then, we used a waterfall plot based on sequencing data from 450k DNA methylation chips in the TCGA project to visually show the relationship between methylation levels and gene subregions (Fig. 2C). After setting the adjusted *P* value at 0.01 and the beta value at 0.25, we obtained a number of CASZ1-related probes which had high levels of methylation including, cg18236877, cg10053369, cg24293689, cg09179249, cg04896832, cg00925802, cg11222217, cg22853713, cg11732134, cg18271964, cg23670372, cg16926809 and cg17158913. Then we visually demonstrated the differences in methylation levels of these probes between tumor tissue and normal tissue and looked into the precise relationships that existed between these probes’ methylation levels and CASZ1 expression in ccRCC (Fig. 2D, E). The findings showed that the methylation level of all probes negatively correlated with CASZ1 gene expression in the 333 ccRCC samples. The strongest negative correlation was found in the CpG island-related probe cg04896832 (*r*=−0.68, *P*<0.001), which was followed by cg18236877 (*r*=−0.66, *P*<0.001), cg10053369 and cg23670372 (*r*=−0.62, *P*<0.01), cg11732134 and cg17158913 (*r*=−0.59, *P*<0.001), cg24293689 (*r*=−0.58, *P*<0.001), cg11222217 and cg22853713 (*P*=−0.57, *P*<0.001), cg09179249 and cg18271964 (*r*=−0.56, *P*<0.001), cg00925802 (*r*=−0.55, *P*<0.001), and cg16926809 (*r*=−0.54, *P*<0.001). Furthermore, upon combining the probe data related to CpG island, the combined outcome showed a correlation value of −0.68 (*P*<0.001), indicating that the low expression of CASZ1 could potentially be attributed to the methylation alteration linked to CpG island. Lastly, we looked into the relationship between the prognosis and the methylation levels of these probes (Fig. 2F). The findings show that the high-methylation subgroup of these probes, which include cg04896832 (*P*<0.0001), cg00925802 (*P*=0.0038), cg11222217 (*P*=0.0116), cg18271964 (*P*<0.0171), and cg16926809 (*P*<0.0001), had a poorer prognosis than the low-methylation subgroup.

Overall, these findings indicated a clear association between CASZ1-related methylation modification and gene regulation as well as the prognosis of RCC patients.

### Explore potential treatment strategies based on different CSAZ1 expression patterns in ccRCC

**Figure 3:**
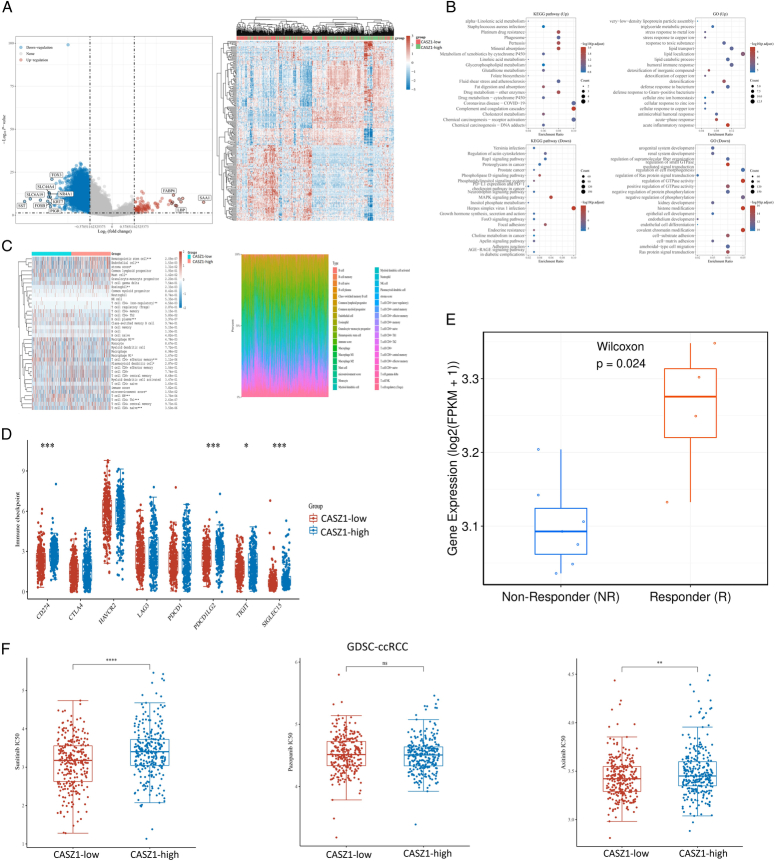
The Castor zinc finger 1 (CASZ1)-low and CASZ1-high subgroups had different enriched functions and pathways and distinct CASZ1 expression patterns could indicate potential therapeutic strategies in clear cell renal cell carcinoma (ccRCC). (A) Differential genes between the CASZ1-low and CASZ1-high subgroups and 50 up-regulated genes and 50 down-regulated genes with the largest differential changes. (B) KEGG pathway enrichment analysis and GO enrichment analysis of genes up-regulated in the CASZ1-low subgroup and genes up-regulated in the CASZ1-high subgroup. (C) Immune cell score in the CASZ1-low and CASZ1-high subgroups and the percentage abundance of tumor-infiltrating immune cells in each sample.*P<0.05, **P<0.01, ***P<0.001. (D) The expression of immune checkpoints in the CASZ1-low and CASZ1-high subgroups. *P<0.05, **P<0.01, ***P<0.001. (E) The difference of the expression of CASZ1 between Non-Responder (NR) and Responder (R). (F) Distribution of Sunitinib, Pazopanib and Axitinib IC50 scores in the CASZ1-low and CASZ1-high subgroups. *P<0.05, **P<0.01, ***P<0.001, ****P<0.0001.

We concentrated on investigating the transcriptome differences between the CASZ1-low and CASZ1-high subgroups in order to identify possible therapeutic approaches for each. To achieve this, we initially compared the differentially expressed genes in the CASZ1-low and CASZ1-high subgroups using TCGA database analysis (Fig. 3A). The findings revealed 101 up-regulated genes and 3774 down-regulated genes in the CASZ1-low subgroup compared to the CASZ1-high subgroup. The KEGG pathway (Up) and GO (Up) enrichment analyses revealed that the majority of the up-regulated genes in the CASZ1-low subgroup were enriched in pathways including “Drug metabolism-other enzymes” and “Complement and coagulation cascades” (Fig. 3B). Furthermore, by comparing immune infiltration and assessing the expression of immunological checkpoints, we investigated the differences in immune state between the groups with high and low CASZ1 expression. Our analysis showed that the CASZ1-high subgroup had a higher percentage of T cell CD8+ naive cells, and that the CASZ1-high subgroups had higher expression levels of immunological checkpoints like CD274 (*P*<0.001), PDCD1LG2 (*P*<0.001), SIGLEC15 (*P*<0.001), and TIGIT (*P*<0.05), compared to the CASZ1-low subgroups(Fig. 3C-D). Then, we conducted data analysis from a pd-1 treatment dataset, RCC-GSE67501_anti-PD-1, using the TIGER tool. The results revealed that patients who responded well to PD-1 therapy exhibited significantly higher CASZ1 expression levels compared to those with poor treatment response (Fig. 3E). Therefore, we deemed that patients with high CASZ1 expression are more suitable for immunotherapy, such as PD-1 pathway blockade therapy.

Then we investigated the potential differences of targeted therapeutic response between the high and low CASZ1 expression subgroups. Our analysis revealed that the sensitivity to tyrosine kinase inhibitor (TKI) drugs which was calculated based on GSDC database, specifically Sunitinib (*P*<0.0001) and Axitinib (*P*<0.01), was significantly higher in the CASZ1-low subgroup compared to the CASZ1-high subgroup, as evidenced by the lower half-maximal inhibitory concentration (IC50) values (Fig. 3F). These findings suggested that ccRCC patients with CASZ1-low expression may be suitable for Sunitini and Axitinib.

## Discussion

We found that three major forms of RCC have aberrant downregulation of CASZ1. Low CASZ1 expression in ccRCC was partially ascribed to DNA methylation modification and was connected with adverse clinicopathological characteristics and a poor prognosis. On the one hand, according to an analysis of immunological state and immune checkpoints in the CASZ1-low and CASZ1-high subgroups, patients with high expression of CASZ1 may benefit from PD-1, PD-L1 pathway blockade therapy. On the other hand, patients who exhibit reduced CASZ1 expression ought to think about traditional anti-angiogenetic treatment. This area needs further investigation and attention

Studies on the expression profiles of CASZ1 in a variety of solid tumors have revealed low expression in neuroblastoma and hepatocellular carcinoma and high expression in malignancies such as epithelial ovarian cancer^21–23^. Previous research has connected CASZ1 malfunction to the genesis and progression of tumors, which may help to explain why decreased CASZ1 expression in ccRCC is associated with poor prognosis and unfavorable clinicopathological characteristics.

Differential regulatory mechanisms in different cancers are suggested by the variability of CASZ1 expression across distinct solid tumors. Nevertheless, no research has looked into CASZ1’s possible regulatory function in RCC. Gene dysregulation can result from a variety of mechanisms, but DNA methylation modification is a crucial one that is strongly linked to carcinogenesis^36^. In this study, we found that the downregulation of CASZ1 in ccRCC may be explained by certain DNA methylation statuses, including hypermethylation of cg18236877, cg10053369, cg24293689, cg09179249, cg04896832, cg00925802, cg11222217, cg22853713, cg11732134, cg18271964, cg23670372, cg16926809, and cg17158913. Our findings suggest that DNA methylation may be a crucial mechanism regulating CASZ1 expression, but this is just a hypothesis that needs to be investigated and validated.

At the moment, the main treatments for ccRCC are anti-angiogenetic therapy and immunotherapy-based immune checkpoint inhibitor (ICI). Certain populations are eligible for these treatments, and biomarkers are being investigated to differentiate between therapy responses^37,38^. Our analysis did not initially demonstrate high immunogenicity in the CASZ1-high subgroup or high angiogenesis in the CASZ1-low subgroup. However, subsequent analysis confirmed that CASZ1 expression status could guide therapeutic decisions for ccRCC patients. In particular, patients with CASZ1-low expression may benefit from anti-angiogenetic medicines, whereas those with CASZ1-high expression should receive priority treatment from ICI-based immunotherapy. Additionally, overexpression of immune checkpoints like CD274, PDCD1LG2, TIGIT, and SIGLEC15 in ccRCC with CASZ1-high expression suggests the importance of combining immune checkpoint biomarkers to predict ICI-based immunotherapy efficacy and guide potential multi-agent immunotherapy decisions. Although these are just speculations based on our research findings and have not yet been validated by clinical trials, we acknowledged that the current evidence is indeed insufficient to demonstrate that the expression of CASZ1 can directly guide the selection of treatment options. This requires larger sample sizes and prospective studies to draw further conclusions. However, our study provides a new perspective for the selection of two treatment options for patients with ccRCC, and we hope it will inspire and provoke further research in the future.

## Conclusions

The results of this investigation shed light on the CASZ1 expression profile in RCC, revealing a significant association between down-regulated CASZ1 and poorer prognosis in ccRCC. Our results suggest that DNA methylation modification may contribute to CASZ1 dysregulation. The CASZ1-high subgroup characterized by high immune infiltration and immune checkpoint expression may determine the more favorable response to PD-1 and PD-L1 pathway blockade therapy. On the other hand, our findings suggest that anti-angiogenetic treatments would be more suitable for the CASZ1-low subgroup. All things considered, our findings imply that CASZ1 expression may be used to forecast the prognosis of ccRCC and guide future treatment approaches depending on unique genetic traits.

## Ethical approval

The studies involving human participants were reviewed and approved by the Ethics Committee of West China Hospital, Sichuan University.

## Consent

The patients/participants provided their written informed consent to participate in this study. Written informed consent was obtained from the individual(s) for the publication of any potentially identifiable images or data included in this article.

## Source of funding

This study was supported by Natural Science Foundation of Sichuan Province (grant nos. 2022NSFSC0712, 2023NSFSC1856) and 1·3.5 project for disciplines of excellence, West China Hospital, Sichuan University (ZYAI24062).

## Author contribution

F.L. and J.L. designed the research; F.L. and J.L. analyzed the data; F.L. wrote the manuscript. J.L. and X.W. supervised the work and reviewed this manuscript. All authors read and approved the final manuscript.

## Conflicts of interest disclosure

The authors have declared that no conflicts of interest exist.

## Research registration unique identifying number (UIN)

Not applicable.

## Guarantor

X.W.

## Data availability statement

Transcriptomic data of ccRCC, pRCC and chRCC tissues and adjacent normal tissues were derived from the TCGA database and are freely available from https://portal.gdc.cancer.gov/. The 3 ccRCC datasets used for the validation were available from GEO http://www.ncbi.nlm.nih.gov/ geo/ under Accession Numbers GSE53757, GSE40435 and GSE66272. Proteomic data of ccRCC tissues and adjacent normal tissues were obtained from the HPA database https://www.proteinatlas.org/.Clinicopathological data, prognostic information, genomic, methylomic and immunomic data were all derived from the TCGA database. Data of drug sensitivity were derived from the GDSC database https://www.cancerrxgene.org/. Our institutional immunohistochemical raw data of this article will be made available by the authors, without undue reservation.

## Provenance and peer review

Not applicable.
